# April Fooled

**Published:** 1873-09

**Authors:** L. B. Brown

**Affiliations:** Sheldon, Ill.


ARTICLE V.—*April Fooled.* By L. B. BROWN, M.D., Sheldon, Ill.

Was called, April 1st, 1873, to visit Mrs. J. W. Smith, a farmer's wife, age 36 years, weight about 150 pounds, of stout build,—(short and thick is a fair expression)—probably of German extraction.

I found the woman in bed, perfectly comfortable, except from fear that all would not be well with her, having been delivered of a child about eight hours before my arrival by the assistance of a midwife—who, by the by, is a very intelligent and successful one—I must say a *rare* specimen of that class as found in the country.

On examination of the abdomen, which particularly attracted my attention, I found it *enormously* distended generally; no pain on pressure, no motion, described by the woman; nor could I detect anything satisfactory. Made examination per vaginam; passed my finger into the os, without difficulty; detected nothing but the cord of the child that had been delivered—made slight tension upon it, with my other hand upon the abdomen—cord easily gave way, and *I was lost.* Does any one suppose the midwife in attendance had been *pulling* at that cord? I suspected and conjectured "muchly"; one thing I thought I knew beyond doubt, to wit, that there must be a retained placenta. Remembering that ergot had been used many times with different results, and having some of Thayer's Fluid Extract, I commenced with twenty drops, repeated every fifteen minutes until four or five doses were given. The woman complained of being dizzy and sick at the stomach; pulse very feeble, surface cold and clammy; vomited; resorted to hot tea and whisky. In about half an hour reaction was sufficiently established, and I was fully satisfied with the pernicious effects of ergot, in that case in particular.

During all this time, two hours or more, there were no uterine pains, no motion, no nothing—but one child, and anxiety.

Determined to introduce my hand into the uterus, I immediately made the effort, and succeeded without difficulty. Found about a hand's length from the os, the back of a child; the vertebræ being my guide, I easily made fast to the inguinal region with my forefinger, and by the aid of a broad towel placed over the abdomen, with an assistant at each end pulling downwards, I delivered the woman of a living child, without any perceptible uterine contractions.

Size of the abdomen not materially diminished; patient comfortable; continued pressure with the towel and introduced my hand again; found sack presenting of large size—was not fully satisfied as to the character and contents; I ruptured the sack, and a very large amount of water escaped, probably two quarts; the abdomen somewhat diminished in size, and to my surprise, the head of another child presented, which I clasped in my hand, and by the aid of the towel as before, delivered the woman of the third living child, without uterine pains.

I now proceeded to prospect for the placenta by introducing my hand, (which was a very easy matter), and, to my utter astonishment and consternation, the head of another child, if possible, presented, which I delivered in the same manner as before, instructing the assistants to pull harder on the towel. Four living children.

During the birth of the fourth child there were slight labor pains.

Now for the afterbirth, with fear but no trembling.

I took the three remaining cords, twisted together slightly, in one hand, introducing the other hand into the uterus and grasped the mass, then gradually but carefully made tension upon the cords, at the same time using other means to excite uterine contractions, which were more forcible in the delivery of the placenta than with the last three children.

The woman was very much exhausted, notwithstanding she had no severe labor pains (owing, no doubt, to the extreme attenuation of the walls of the uterus). I was fearful syncope might overtake her, from the removal of the compression to which the abdominal veins had been subjected. A broad bandage was immediately fixed around the abdomen tightly, and mild stimulants administered. No trouble in maintaining a contracted state of the uterus.

The children: all girls, perfectly formed, and of fair size, very similar in shape, rather long and slender; round heads, well ossified; aggregate weight eighteen pounds—two weighing five pounds each, and two weighing four pounds each.

The placenta was quadrilateral in form, twelve or fourteen inches square, and of the usual thickness. The cords were regularly attached, about seven inches apart. I did not weigh the placenta, —probable weight six or seven pounds.

Taking the aggregate, children, placenta, and liquor amnii, which could not be acurately estimated, though in my opinion not less than three quarts, it will require some stretch of the imagination to comprehend the appearance and condition of the woman before and during labor.

In 129,172 births in the Lying-in Hospital of Dublin, there were 29 cases of triplets and one of quadruple birth.

From British, German and French records, the following is reported: 666,424 cases, 8,006 twins, 87 triplets.

This extremely rare occurrence of quadruple gestation, and the still greater rarity of four living, perfectly formed, fair sized children, is my excuse for reporting the above case.

The children are now (July 17) three and one-half months old, and in a prosperous condition, weighing respectively 11, 10, 10, 9 pounds; total, 40 pounds.

